# Spontaneous Bleeding in Pancreatitis Treated by Transcatheter Arterial Coil Embolization: A Retrospective Study

**DOI:** 10.1371/journal.pone.0072903

**Published:** 2013-08-20

**Authors:** Veit Phillip, Sebastian Rasch, Jochen Gaa, Roland M. Schmid, Hana Algül

**Affiliations:** 1 II. Medizinische Klinik und Poliklinik, Klinikum Rechts der Isar, Technische Universität München, München, Germany; 2 Institut für Röntgendiagnostik, Klinikum Rechts der Isar, Technische Universität München, Munich, Germany; University of Munich, Germany

## Abstract

**Background/Objectives:**

A rare, but life-threatening complication in pancreatitis is a spontaneous bleeding from intestinal vessels with or without previous formation of (pseudo-) aneurysms. And yet, the optimal diagnostic and therapeutic strategies remain unclear.

**Methods:**

We performed a retrospective analysis of all patients with pancreatitis and intraabdominal bleeding at a German tertiary referral center between January 2002 and December 2012.

**Results:**

Bleeding occurred in <1% (14/3,421) of patients with pancreatitis. Most involved vessels were arteria lienalis, arteria gastroduodenalis, and arteria pancreaticoduodenalis. All bleedings could be stopped by transcatheter arterial coil embolization. Recurrent bleeding after coil embolization occurred in 2/14 (14%) patients.

**Conclusions:**

In cases of intraabdominal hemorrhage in patients with pancreatitis, transcatheter arterial coil embolization should be considered as the first interventional procedure.

## Introduction

Acute pancreatitis (AP) is a common disease with an incidence of 19.2 to 42.8/100,000 person-years and a mortality of nearly 5% [1–3]. However, the severe form of AP, including exacerbation of pre-existing co-morbidities, local complications such as acute peripancreatic fluid collection, pancreatic pseudocysts, acute pancreatic and peripancreatic necrosis, walled-off necrosis, and persistent organ failure occurs in 20-30% of all cases and is associated with a high mortality of up to 50% [4–7].

One of the complications in pancreatitis is spontaneous bleeding from intestinal vessels with or without previous formation of (pseudo-) aneurysms. These life-threatening bleedings are rare complications occurring in about 1-5% of patients with AP or chronic pancreatitis (CP) [8–13]. Pathophysiologically, progressive inflammation within the pancreas and the surrounding tissue promotes the arrosion of vessels and formation of (pseudo-) aneurysms by extravasation of proteolytic enzymes, formation of pseudocysts, extensive necrosis, and abscesses [14,15]. These processes can be aggravated by endoscopic, radiologic or surgical necrosectomy. While one study showed no bleeding complications neither after endoscopic transgastric nor after surgical necrosectomy [16], in numerous other studies high rates of intraabdominal bleeding after endoscopic, radiologic, or surgical necrosectomy were reported [17–19]. A further study compared different necrosectomy treatments and showed bleeding rates of 17-26% for the different approaches [20]. In the postoperative setting after partial pancreatectomy, intraabdominal abscess formation and pancreatic fistula are the most common cause for vessel arrosion and bleeding [21,22].

Some small case series and review articles describe different diagnostic approaches including contrast enhanced computed tomography (CE-CT) and digital subtraction angiography (DSA) for detection of spontaneous bleeding [23,24]. Different therapeutic strategies including transcatheter arterial embolization (TAE), coiling, stenting, operative ligation of bleeding vessels, operative intraabdominal packing, or pancreatectomy are therefore proposed [8,25,26]. And yet, the optimal diagnostic and therapeutic strategy remains unclear.

In this retrospective analysis, we report a centre-specific experience of transcatheter arterial coil embolization for intraabdominal hemorrhage in patients with acute or chronic pancreatitis.

## Methods

The study was approved by the local ethics committee (Ethikkommission der Fakultät für Medizin der Technischen Universität, München). Written consent was specifically waived by the approving institutional review board. We analyzed the administrative diagnosis database for patients with pancreatitis (International Classification of Diseases (ICD)-10 code K85 and K86) between January 2002 and December 2012. A database including all patients undergoing intraabdominal TAE was matched to the patients with pancreatitis.

### Statistical analyses

All statistical analyses were performed using IBM SPSS Statistics 20 (SPSS Inc, Chicago, Illinois, USA). If appropriate, descriptive data are presented as mean ± standard deviation or median, range, and interquartile range (IQR). The Mann–Whitney U test was used to compare continuous data of patients with and without intraabdominal bleeding. All statistical tests were 2-sided and performed in an explorative manner on a 5% significance level.

## Results

### Patients’ characteristics

We identified 7,382 cases in 3,421 patients and registered 1,302 patients with AP and 2,119 patients with CP treated at our hospital during the study period. Due to reasons of privacy data protection, personal data are available for only 4,529 (61%) patients. There was no statistically significant difference of age in patients with intraabdominal hemorrhage compared to patients without intraabdominal hemorrhage (median, 60 years (range, 40-77 years; IQR, 52-69 years) vs. 58 years (range 12-94 years; IQR 47-68 years; p=0.379)). Fourteen patients with intraabdominal hemorrhage due to acute (n=11), chronic (n=1), or an acute attack of chronic (n=2) pancreatitis were identified. Regarding patients with AP, 10/11 (91%) experienced their first attack of AP, and 8/11 (73%) developed a necrotizing pancreatitis. Patients’ characteristics and etiology of AP are shown in tables 1 and 2, respectively.

**Table 1:** Patients’ characteristics (n=14).

Sex (male/female)	10/4
Age, years	60; 40-77; 52-69
Body weight, Kg	87; 60-120; 74-105
Body height, cm	180; 150-184; 159-181
Body mass index, Kg/m^2^	27.8; 22.9-32.8; 23.4-32.5
Duration of hospital stay, days	39; 3-368; 13-131
Duration of intensive care treatment, days	11; 0-368; 1-37
Time AP to bleeding, days	42; 4-213; 17-104
Time CP to bleeding, month	60

Data are presented as median, range, and interquartile range.

Body weight, body height, and BMI are available for 7/14 (50%) patients.

**Table 2:** Etiology of acute pancreatitis (n=11).

alcoholic	3 (27%)
biliary	3 (27%)
post-ERCP	2 (18%)
idiopathic	2 (18%)
pancreas divisum	1 (9%)

ERCP, endoscopic retrograde cholangiopancreatography

Nine out of 14 patients (64%) required intensive care unit (ICU) treatment. Mechanical ventilation was necessary in 6/14 (43%) patients, 5/14 (36%) developed a circulatory failure requiring catecholamine therapy, and acute renal injury according to the Acute Kidney Injury Network occurred in 6/14 (36%), 4 of them underwent dialysis [27].

### Time point of hemorrhage

In patients with AP, the median duration from onset of pain to arrosion bleeding was 42 days (range, 4-213; IQR 17-104). The time between onset of pain and bleeding complication in the two patients with an acute attack of CP was 5 and 14 days, respectively. Duration from initial diagnosis of CP and intraabdominal bleeding in the patient without an acute attack was about 60 months.

### Involved vessels

**Figure 1:**
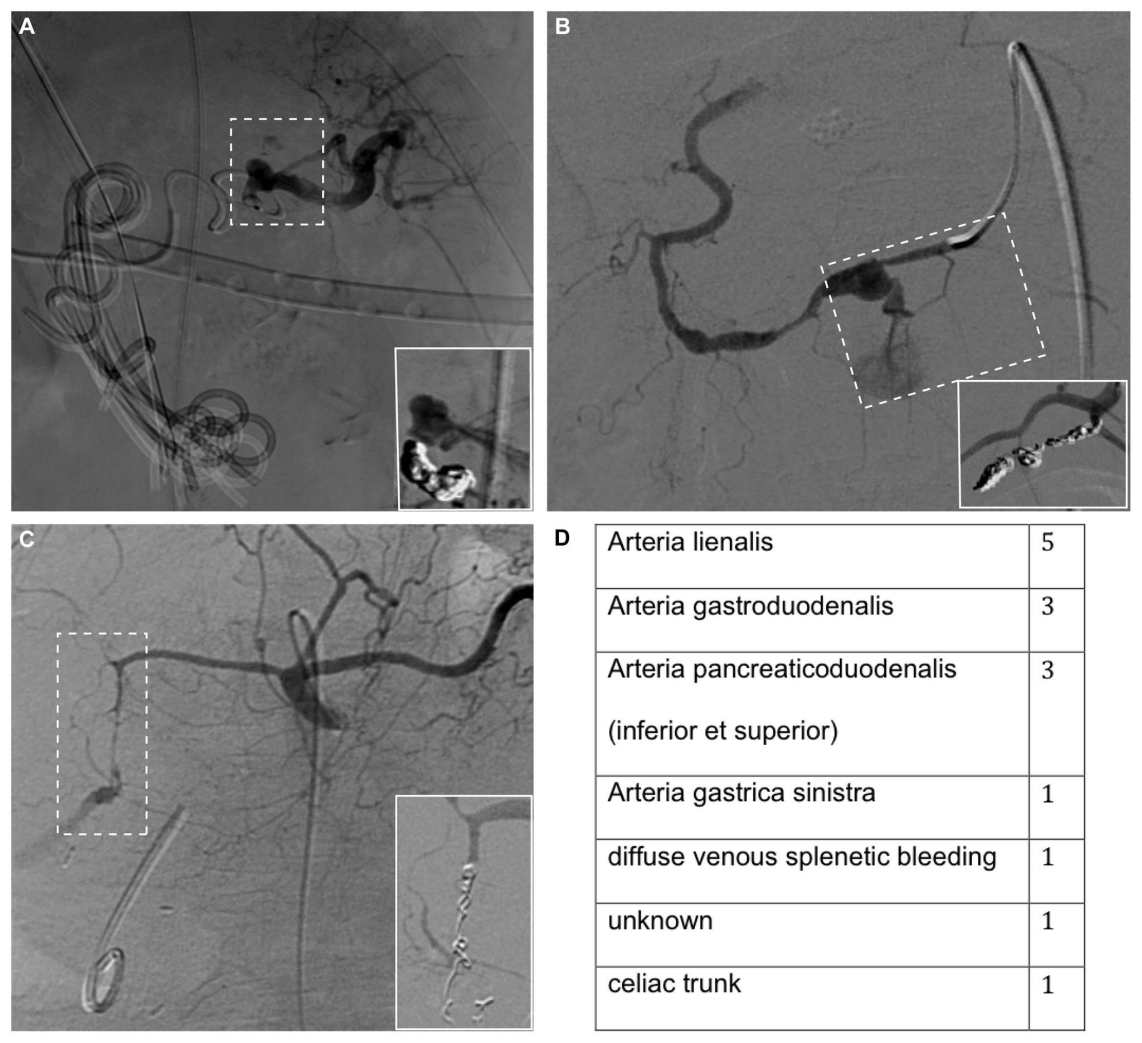
Illustration of bleeding vessels before and after transcatheter arterial coil embolization. Digital subtraction angiography showing bleeding of arteria lienalis (A), arteria pancreaticoduodenalis inferior (B), and arteria gastroduodenalis. Inlets demonstrate transarterial coil embolization. The localizations of intraabdominal bleedings are shown in the table (D).

In 13/14 (93%) cases, the source of bleeding was an arterial blood vessel. Only one case showed diffuse venous splenic bleeding. Localizations of bleedings are shown in Figure 1.

In 8/14 (57%) cases, a true or false bleeding aneurysm was detected by CE-CT or arteriography.

### Diagnostic

In 13/13 (100%) cases, the bleeding could be located by CE-CT. In one case, no CE-CT was performed before DSA. In contrast, an active bleeding could be seen in only 8/14 (57%) during DSA.

### Therapy

All but one (13/14; 93%) bleedings could be stopped by transarterial coil embolization. In the remaining case, neither the bleeding diagnosed using CE-CT nor an aneurysm could be seen during DSA and therefore no coiling was performed. According to the localization of the bleedings, the most coiled artery was arteria lienalis (7x) followed by arteria gastroduodenalis (3x), arteria pancreaticoduodenalis (3x), arteria gastrica sinistra (1x), arteria hepatica sinistra (1x), and arteria hepatica communis (1x). The median number of coils used per patient was 5 (range 0-16; IQR, 4-10).

### Risk factors/Interventions before bleeding

Only one out of 14 patients (7%) had surgery before intraabdominal hemorrhage. However, in 8/14 (57%) patients, an intraabdominal catheter was placed in order to drain infected necroses. In 6/8 (75%) of theses patients, a percutaneous access was used and in 5/8 (63%) a transgastric drainage was placed. The median number of percutaneous (range 1-6) and transgastric drainages (range 1-5) was 2 in both groups.

### Outcome

Five out of 14 patients died before discharge from the hospital. Accordingly, hospital mortality was 36%. Among the 9 patients treated at an ICU, 3 (33%) died before transfer to normal ward. The median time between bleeding and death in all patients was 15 days (range, 2-152 days; IQR, 3-141). Only one patient died as a consequence of intraabdominal bleeding.

Recurrent bleeding after coil embolization occurred in 2/14 (14%) patients. Time between coiling and recurrent bleeding was 0 and 9 days.

## Discussion

Arrosion bleeding of intraabdominal vessels is a severe complication in acute and chronic pancreatitis. Based on our data the choice of successful treatment in such cases is immediate transcatheter arterial coil embolization (TAE).

Symptoms of intraabdominal hemorrhage in patients suffering from pancreatitis are abdominal pain, bleeding from drainages, hemorrhagic shock and decrease of hemoglobin [28]. In case of at least two of these symptoms, immediate diagnostic by CE-CT or arteriography should be performed [22,29]. All but one bleeding in our patients derived from an arterial vessel. Due to the proximity to the pancreas, the most involved vessel was arteria lienalis, followed by arteria gastroduodenalis and arteria pancreaticoduodenalis [14].

In case of intraabdominal bleeding diagnosed by CE-CT or angiography, an interventional angiography and stenting [30,31], coiling [32], or embolization [33] should be achieved [15]. Embolization of hemorrhage due to arrosion of vessels showed good results in small series of postoperative patients [34] and patients with pancreatitis [26,32]. According to a few case series, TAE seems to be effective in the management of pseudoaneurysms and hemorrhage and may result in temporary or permanent control of acute hemorrhage [14,15,26]. Conversely, laparotomy and ligation of bleeding vessels may be difficult due to adhesions and tissue friability in such patients [35,36]. Laparotomy for therapy of bleeding should be considered only in hemodynamic instable patients or unsuccessful coiling [14,15,22,29,35], while surgical packing is the procedure of choice in diffuse venous intraabdominal bleeding [8]. In most cases, it seems that TAE is sufficient to control bleeding complications. Even in our study no primary surgical approach was required to stop bleeding, although 5/14 (36%) were in instable condition, requiring periinterventional catecholamine therapy. In two patients, a relapse of the bleeding occurred. While one patient underwent a further TAE with additional coiling, bleeding in the second patient could only be stopped by ligation of the splenic artery after laparotomy.

**Figure 2:**
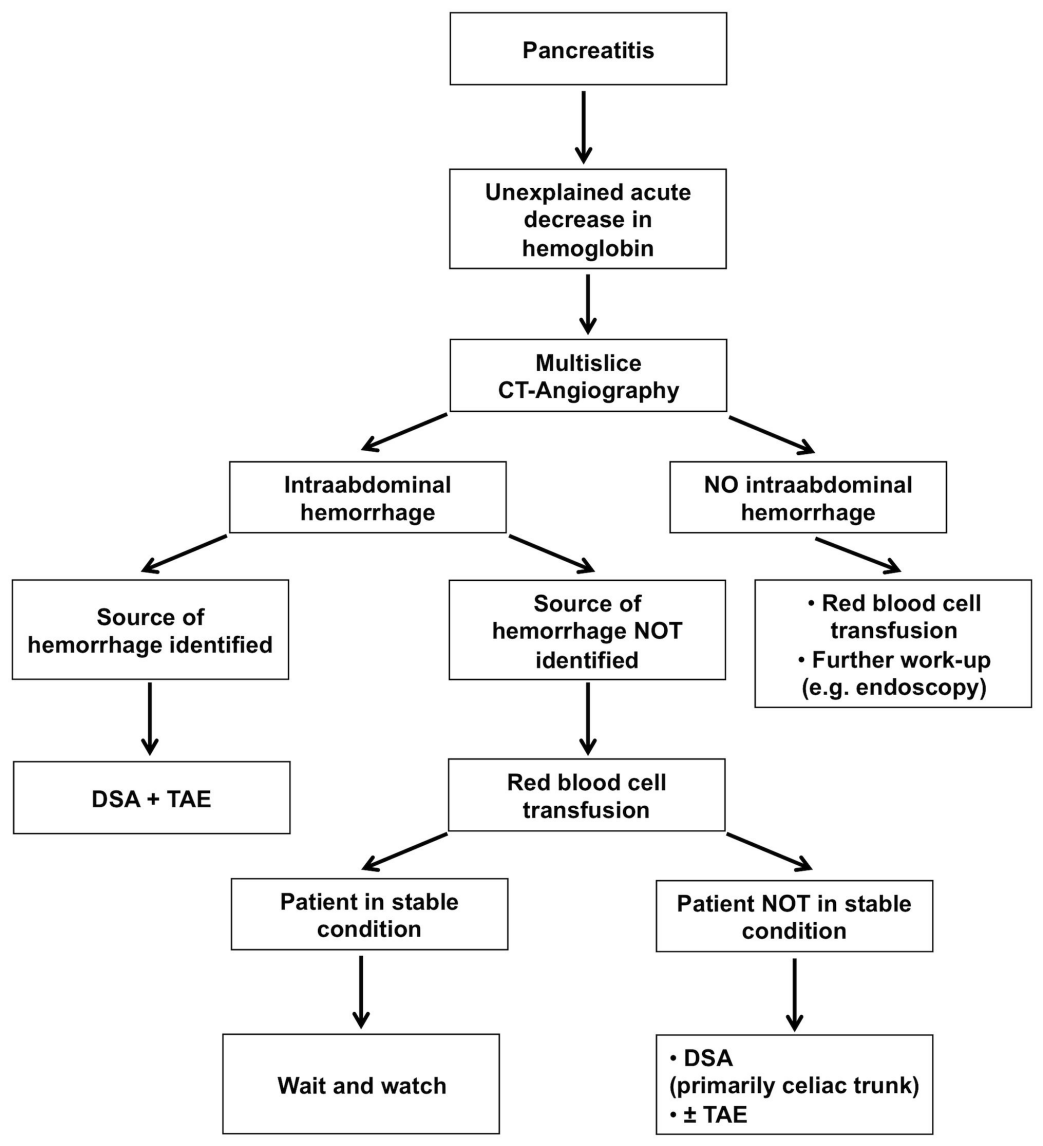
Algorithm for the management of intraabdominal hemorrhage in pancreatitis. CT, computed tomography; DSA, digital subtraction angiography;. TAE, transcatheter arterial embolization.

Our data and others identified arteries arising form the celiac trunk as most frequent sources of bleeding (Figure 1) [14]. Given normal anatomy, immediate angiography of the celiac trunk should be performed in patients in unstable condition and not identified source of intraabdominal hemorrhage (Figure 2).

Patients with severe AP have in any event a high mortality of up to 48% [6]. Mortality seems to be even higher in patients with AP complicated by intraabdominal hemorrhage of up to 60% [14]. In some studies a three fold higher probability of a fatal outcome in patients with severe AP and hemorrhagic complications than in those with severe AP without bleeding complications was reported [8]. However, others suggest that hemorrhagic complications per se have little influence on mortality [9]. Bleeding complications seem to reflect rather the severity of the disease than being the direct cause of mortality [9,37]. In our study, we observed a hospital mortality of 36%, which is considerably lower than described before [14]. One patient died as a consequence of intraabdominal bleeding, 3 patients died from septic complications and one patient died after transmission to another hospital for unknown reasons. Advances in interventional radiology over the last years potentially account for the lower mortality in our study.

In more than half of the patients (57%) intraabdominal catheter was placed to drain infected necrosis prior to bleeding. Indeed, in addition to pseudocysts, necroses and abscesses, intraabdominal drainages have been associated with increased risk for intraabdominal hemorrhage [37]. Mechanistically, percutaneous or transgastric drainages may therefore cause pressure necrosis and erosion of vessels thus provoking intraabdominal bleeding.

In cases of intraabdominal hemorrhage in patients with pancreatitis, TAE should be considered as the first interventional procedure. Based on the widely spread availability and minimal invasiveness of DSA as well as the improved technical capabilities of interventional radiology, we therefore consider TAE as a central component in the proposed algorithm (Figure 2).
